# In-situ Isotopic Analysis at Nanoscale using Parallel Ion Electron Spectrometry: A Powerful New Paradigm for Correlative Microscopy

**DOI:** 10.1038/srep28705

**Published:** 2016-06-28

**Authors:** Lluís Yedra, Santhana Eswara, David Dowsett, Tom Wirtz

**Affiliations:** 1 Advanced Instrumentation for Ion Nano-Analytics (AINA), MRT Department, Luxembourg Institute of Science and Technology (LIST), 41 rue du Brill, 4422 Belvaux, Luxembourg

## Abstract

Isotopic analysis is of paramount importance across the entire gamut of scientific research. To advance the frontiers of knowledge, a technique for nanoscale isotopic analysis is indispensable. Secondary Ion Mass Spectrometry (SIMS) is a well-established technique for analyzing isotopes, but its spatial-resolution is fundamentally limited. Transmission Electron Microscopy (TEM) is a well-known method for high-resolution imaging down to the atomic scale. However, isotopic analysis in TEM is not possible. Here, we introduce a powerful new paradigm for *in-situ* correlative microscopy called the Parallel Ion Electron Spectrometry by synergizing SIMS with TEM. We demonstrate this technique by distinguishing lithium carbonate nanoparticles according to the isotopic label of lithium, viz. ^6^Li and ^7^Li and imaging them at high-resolution by TEM, adding a new dimension to correlative microscopy.

Isotopic analysis is fundamental in nearly all branches of science. In the biological sciences, isotopes are used as labels in a diverse array of topics such as in the investigation of metabolism in plants^1^, humans^2^, animals^3^,^4^,^5^ and microorganisms^6^,^7^. Isotopes are analyzed in archaeology for dating fossilized material^8^,^9^. In the physical sciences, isotopes are used as powerful markers in a wide range of research studies to observe and analyze various physical phenomena such as diffusion^10^,^11^ and corrosion^12^. In astrophysics and planetary sciences^13^,^14^, isotopic analysis is used to accurately study for example, the early stages of the formation of planets^13^. Isotope analyses are also critical in geochronology^15^ and geochemistry^16^,^17^. Technological innovations also rely on analyzing isotopes such as in the research of molecular sieves^18^ and energy-storage materials^19^.

Despite the overarching significance of isotopic analysis across the full spectrum of science, a singular method for high-resolution nanoscale isotopic imaging is lacking due to fundamental physical limitations. A general strategy to overcome the limitations of individual characterization techniques is to combine and correlate different techniques to obtain complementary information about the sample. Until now, such correlative microscopy methods have been primarily based on combining photon-based techniques (e.g. visible light^20^, X-rays^21^) with electron microscopy. Correlation with light microscopy is particularly interesting in biological sciences as it is compatible with live-cell imaging, unlike charged-particle (electron, ion) microscopy. Although these multimodal techniques are very powerful for multi-scale imaging, they are not capable of imaging isotopic distribution, nor giving high-sensitivity analytical information.

The most common technique used for imaging isotopic distribution is based on Secondary Ion Mass Spectrometry (SIMS). The basic principle of this technique consists in using primary ions to sputter the sample and subsequently collecting and mass-analyzing the secondary ions that are ejected from the sample^22^. Owing to its excellent sensitivity, high dynamic range and ability to distinguish isotopes, SIMS is a very powerful tool for scientific research. However, the lateral resolution achievable with SIMS is fundamentally limited due to several factors. The primary limitation to the spatial resolution in SIMS is due to the lateral dimension of the collision cascade. For typical energies of the primary ion beam, the diameter of the area emitting secondary ions around for instance, Ga^+^ impacts ranges between 8 and 10 nm (FW50)^23^. Furthermore, as SIMS is based on sputtering of sample by a primary ion beam, there is a physical limit to the smallest feature size that can be analyzed due to the necessity of acquiring a secondary ion signal of reasonable signal to noise ratio from this feature before it is sputtered away^24^,^25^. This smallest detectable feature size depends in particular on the concentration of the species to be detected within this feature and the ionization rate of the sputtered atoms^23^,^25^. These severe fundamental limitations preclude the application of the technique to unravel the structural and chemical details including isotopic information at scales ranging from sub-nanometer to a few nanometers.

Transmission Electron Microscopy (TEM) is a well-established technique for high resolution imaging down to the atomic scale and generally does not suffer from a sputtering-limited resolution except in a small minority of cases^26^. TEM also allows nanoscale crystallographic analyses based on electron diffraction. The traditional analytical tools associated with TEM are the Energy-Dispersive X-ray Spectroscopy (EDX) and the Electron Energy-Loss Spectroscopy (EELS). These are powerful techniques for elemental analysis and to probe the local electronic and optical properties (EELS)^27^,^28^, but they do not yield any isotopic information.

In this report, we introduce a new paradigm of correlative microscopy combining *in-situ* TEM and SIMS called the Parallel Ion Electron Spectrometry (PIES). By synergizing the strengths of these complementary techniques, we demonstrate that the isotopic analysis can be carried out at unprecedented detail with the spatial resolution offered by TEM. The *in-situ* combination introduced here offers several key advantages such as (i) TEM can be performed first to find interesting nanostructures and then SIMS carried out on those pre-selected features, (ii) conversely, SIMS can be performed first to screen and identify isotopic hotspots and then TEM is used for high-resolution imaging of the hotspots, (iii) TEM and SIMS can be done iteratively and rapidly without the need for sample transfer and thereby avoiding sample modification and contamination and (iv) artefacts associated with SIMS image distortion are corrected directly and accurately with the help of TEM imaging.

The schematics and a photo of the PIES instrument are shown in Fig. 1. The octagon and the pole-pieces of a FEI Tecnai F20 TEM with TWIN objective lens were redesigned to accommodate the FIB and the SIMS such that both the columns are at 68° angle with respect to the optic axis of the TEM. The FIB is a FEI Magnum with monoisotopic ^69^Ga^+^ primary ion source. The SIMS system is based on a compact high-performance double-focusing magnetic sector that was developed in-house. A specially developed TEM sample holder which can be biased to high-voltage (±5 kV) is used to create an electric field to efficiently collect the secondary ions. The goniometer of the TEM can be used to tilt the sample holder by 68° such that the SIMS extraction nozzle is perpendicular to the sample surface thus maximizing the collection of the secondary ions. In this configuration the FIB is at 45° to the sample surface. Additional basic engineering aspects of the instrument development are discussed elsewhere^23^. The SIMS image resolution was evaluated to be sub-60 nm which is comparable to the performance of the state-of-the-art standalone Cameca NanoSIMS instrument^29^. The TEM, despite the modification of the pole-pieces and possible external stray magnetic field originating from the mass spectrometer, retained the pre-modification performance of sub-1.5 Å lattice resolution at 200 keV. Further details are provided in the Methods section.

To demonstrate the full potential of the PIES methodology, lithium carbonate (Li_2_CO_3_) was selected for this study. Lithium exists naturally in two stable isotopic states: ^6^Li and ^7^Li. The natural abundances of ^6^Li and ^7^Li are 7.5% and 92.5%, respectively. Three kinds of Li_2_CO_3_ nanoparticles were analyzed: (i) natural Li_2_CO_3_ (ii) isotopically enriched variant ^6^Li_2_CO_3_ (isotope purity: 95 atomic % ^6^Li) and (iii) a physical mixture of the previous two. Specific details pertaining to sample preparation are available in the Methods section. The goal of this investigation was to distinguish nanoparticles *in-situ* according to their isotopic state and to image them at high resolution by PIES. The demonstration will pave the way for a spectrum of diverse investigations in nearly all domains of science.

The isotopic analyses with SIMS were performed with the Ga^+^ primary ion beam at an impact energy of 26 keV (FIB operated at 30 kV, sample biased to +4 kV) with a probe current of 50 pA. The SIMS images were acquired with 512 × 512 pixels with a probe dwell time of 0.2 ms such that the time for total image acquisition was 52 s. The TEM images were obtained in the standard bright-field (BF) mode at 200 kV operating voltage.

Figure 2A shows two mass spectra obtained from samples containing (i) only natural Li_2_CO_3_ (in red) and (ii) only isotopically enriched variant ^6^Li_2_CO_3_ (in green). By integrating the mass peaks of ^6^Li and ^7^Li, the abundance of ^6^Li was found to be 8.9% in natural Li_2_CO_3_ and 96.8% in the isotopically enriched variant ^6^Li_2_CO_3_. This is in good agreement with the globally expected average values mentioned previously. The acquisition of the mass spectra is a necessary intermediate step to precisely position the secondary ion detectors in the focal plane of the mass spectrometer for subsequent SIMS imaging.

The SIMS images obtained from sample (iii) containing a physical mixture of both the natural and the isotopically enriched nanoparticles are shown in Fig. 2. The Fig. 2B,C correspond to SIMS images of ^6^Li^+^ and ^7^Li^+^ secondary ion distributions, respectively. The BF-TEM image of the same area is shown in Fig. 2D for comparison. An overlay of the two SIMS images is shown in Fig. 2E. Comparing Fig. 2D with Fig. 2E, the particles can be readily indexed according to the isotopic label.

In order to correlate the structural details seen in the BF-TEM image with the SIMS images of ^6^Li^+^ and ^7^Li^+^ shown in Fig. 2, an overlay of these three images is necessary. Methodologically, for an accurate overlay of the SIMS and TEM images, the Fields-of-View (FoV) have to be matched precisely. The geometrical configuration of PIES (cf. Fig. 3A) imposes *a priori* that the FoV obtained by SIMS and TEM will not be exactly identical. The primary ion column is placed at an angle of 45° to the sample surface so that the SIMS nozzle can be perpendicular to the sample surface to obtain highest efficiency in the collection of the secondary ions. This geometry implies two main consequences: (i) The nominal x:y ratio of the sides of SIMS images is 1: √2 (neglecting the effect of the sample bias on the incidence angle of the primary ions) due to the projection of a square raster area transforming into a rectangle on the sample with the elongated side given by 1/sin(45°) and (ii) topography effects that can cause ‘shadowing’. These effects are illustrated in Fig. 3.

While the geometric effect is the primary factor distorting the SIMS image, other angular deviations such as minor image-rotation (θ) and shear (α) need to be also quantified and corrected prior to the image overlay. These parameters have to be evaluated for each instrumental setting such as the FIB raster area, magnification-dependent TEM image-rotation and the polarity and magnitude of the sample bias. Whereas in a stand-alone SIMS instrument the distortion in SIMS images is not easily evaluated, the PIES methodology directly reveals the exact size and shape of area from which SIMS images were acquired simply due to the contrast in the BF-TEM image taken at a lower magnification (cf. Fig. 3B). With this information, important parameters for image overlay such as the image rotation, elongation and shear can be reliably and rapidly quantified and corrected. This is an additional advantage in the PIES methodology.

Standard procedures involving geometric affine transformation^30^ were applied to correct the image-distortion present between the SIMS and TEM images. The standard matrix transformation for an image in (x, y) coordinates onto new (x′, y′) coordinates is given by:


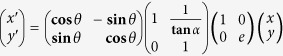


where every *x* and *y* position in the SIMS image corresponds to *x*′ and *y*′ position in the TEM image. The elongation factor *e* was 1.38 (cf. aforementioned geometric factor √2), the shear (α) 7.9° and the rotation (θ) 13.3° for the TEM image shown in Fig. 3B.

After the image transformations, the PIES image as shown in Fig. 4A is then obtained by fusing the SIMS and TEM images. This was carried out using the standard Pan-Sharpening algorithm^31^. The principle of this method is as follows. The RGB (red-green-blue) image of a SIMS image is converted to an HSV (hue-saturation-value) image. The ‘value’ parameter is then merged with the intensity of the higher resolution TEM image and converted back to RGB image such that the color and the intensity of the PIES image carry the chemical and structural detail respectively. Note that direct overlay of the BF-TEM image (contrast as-acquired) with the SIMS data would result in most of the colour falling onto black areas where it would be difficult to visualize. For this reason, the BF-TEM image with inverted contrast is overlaid with the chemical information in SIMS as shown in Fig. 4A. The precision of the co-registration is limited by the resolution of SIMS images, i.e. ~60 nm.

Selected SIMS hotspots highlighted in Fig. 4A are subsequently imaged at high resolution by TEM and are shown in Fig. 4B–D. The corresponding SIMS images are magnified to match the TEM fields-of-view and are shown in Fig. 4E–G. A comparison of the TEM and SIMS image-pairs immediately reveals the unique and powerful complementarity enabled by the PIES methodology: the TEM images provide fine structural details to the SIMS images and in turn, the SIMS images provide isotope-specific information to the TEM images indicated here by red (^7^Li^+^) and green (^6^Li^+^) arrows. The synergistic methodology of PIES thus demonstrates the potential of high-resolution imaging together with the ability to distinguish isotopes.

In the workflow wherein SIMS imaging is done first to find interesting chemical hotspots followed by TEM imaging, minor artifacts originating from the SIMS pre-screening inevitably appears in the TEM images. During the initial localization of the RoIs with SIMS, large Fields-of-View are scanned such that the distance between the successive spots is larger than the spot size, resulting in arrays of small bright spots such as in the background of BF-TEM image in Fig. 4B. It is often not feasible to obtain the high resolution TEM images without knowing where the chemical hotspots would be. However, in the workflow wherein TEM imaging is done first to find interesting structures followed by SIMS imaging, there is no such limitation.

Some of the potential pitfalls inherent to SIMS are also applicable to the PIES technique and it is imperative to discuss them briefly here. The foremost aspect is the effect of surface topography^32^. The pitfalls that arise due to sample topography are (i) unequal sputter yield dependent on the local angle of incidence of the primary ion beam (ii) shadow effect: as the primary ions arrive at 45° incident angle, the shadowed area (seen more clearly in the TEM image in Figs 2D and 3B) will not be sputtered and (iii) local distortion in the extraction electric field due to sample topography can be detrimental to the collection efficiency of secondary ions. However, these issues do not hamper the identification of the nanoparticle by isotopic labeling. Ideally, samples with flat surface such as thin ultramicrotome sections used in biological TEM samples are the best to avoid this pitfall. For samples where there is an evolution in topography due to SIMS, other elaborate approaches such as correlating with Atomic Force Microscopy (AFM) imaging need to be involved^23^,^33^. Re-deposition of sputtered ions and neutral atoms (cf. large particles in Fig. 2B,C) onto adjacent area of the sample can lead to spatial misattribution. A solution to this issue is available in the PIES methodology wherein the contrast in TEM image (cf. Fig. 2D) taken before and after SIMS analyses can give useful insights for correct interpretation depending on the correlative workflow chosen. Another issue to be highlighted is the inherent difficulty in quantification of SIMS results. This limitation is also applicable in PIES. Rigorous quantification will require reference samples of known concentrations in similar matrix as that of the investigated sample. Nevertheless, correlation with other quantitative analytical tools present in TEM such as EDX or EELS, although not isotope-specific, can be helpful for elemental quantification.

In addition to isotope-specific imaging, another attractive feature of the PIES methodology is the high-sensitivity high-resolution analysis capability. Traditional analytical tools in TEM such as EDX or EELS do not have the high sensitivity in the ppm level which is possible with SIMS if sufficiently large voxels are probed. Therefore, imaging of the distribution of trace concentrations of elements, for instance, sub-millimolar concentration of elements in biological material or dopants in semiconductors at high spatial resolution is also possible with PIES methodology. In this context, it is relevant to discuss the ionization efficiency and its implications for the detection limit of SIMS. Indeed in commercial SIMS instruments (e.g. Cameca) Cs^+^ and O^-^ primary ions are routinely used because the integration of these reactive species into the sample leads to high ionization efficiencies of sputtered atoms and molecules. Although Ga^+^ is relatively less reactive (i.e. poorer ionization efficiency), it offers higher lateral resolution due to high-brightness Ga^+^ ion sources. The Useful Yield (UY) with Ga^+^ primary ions for positive secondary ions (SI) was found to be nearly the same as with O_2_^+^ primary sources for oxygen-containing samples such as oxides^34^. In other cases, the UY was 1 to 2 orders of magnitude worse than with O_2_^+^ primary sources^34^. Likewise, the negative SI yields with Ga^+^ primary have been found to be 3–4 orders of magnitude worse than with Cs^+^ primary sources^34^. Fortunately, for both positive and negative SI, reactive gas flooding has been shown to help enhance the ionization efficiency by up to 2 orders of magnitude for Cs^0^ flooding^35^,^36^ and by 1 order of magnitude for O_2_ flooding^37^. The reactive gas flooding has been shown to be successful even for less reactive primary ions of noble gases^38^. Indeed there are ports available around the TEM sample chamber which will be used in the future to deliver reactive gases locally to improve the ionization yields and thereby enhance the detection limit.

In conclusion, we have introduced a new paradigm for correlative microscopy using *in-situ* combination of TEM and SIMS, called PIES. We have demonstrated the potential of the PIES method for multimodal nanoscale isotope analysis with the example of ^6^Li and ^7^Li. With the PIES approach, the sputtering-limited resolution of the SIMS imaging mode is circumvented by complementing the SIMS data with the high-resolution imaging capability in the TEM mode. This new method can be applied in all domains of science ranging from life sciences to physical sciences where analyzing isotopes at nanoscale is vital to push the frontiers of knowledge.

## Methods

### Performance and Operation of the PIES Instrument

To evaluate the instrumental performance of SIMS and the TEM post-modification, SIMS and TEM images were acquired and the spatial resolution were determined according to standardized procedures. A SIMS image of ^7^Li^+^ obtained from a lithium titanate sample is shown in the supplementary Fig. S1-A. The image was acquired with 100 pA Ga^+^ primary ion current at an impact energy of 26.5 keV (FIB operated at 30 kV, sample biased to +3.5 kV). A line profile taken perpendicular to an edge of the sample (indicated by an arrow) is shown in Fig. S1-B. Using the standard IUPAC definition of 16–84% intensity change, the resolution is evaluated to be sub-60 nm. This value is similar to the state-of-the art standalone Cameca NanoSIMS instrument^29^. The TEM imaging performance was evaluated with the standard Au nanoparticles on amorphous carbon reference sample. The HR-TEM image taken at Scherzer defocus with 200 keV electrons is shown in Fig. S1-C. The lattice fringes of the (111) Au crystal are clearly visible. The Fast Fourier Transform (FFT) of the image is shown in Fig. S1-D. The spatial frequencies corresponding to (222) lattice planes are visible indicating sub-1.5 Å lattice resolution. Thus the modification of the TEM objective lens pole-pieces and the presence of possible stray magnetic fields originating from the mass spectrometer have no noticeable effect on the performance of TEM. Note that during SIMS analyses, the electromagnetic objective lens of the TEM is turned off to avoid interference on the SIMS optics. On the FIB side, however, the use of monoisotopic ^69^Ga^+^ primary ion source was necessary to avoid the primary probe splitting into two separate probes of ^71^Ga^+^ and ^69^Ga^+^ under the residual magnetic field of the objective lens of the TEM.

### Sample Preparation

**Figure 2:**
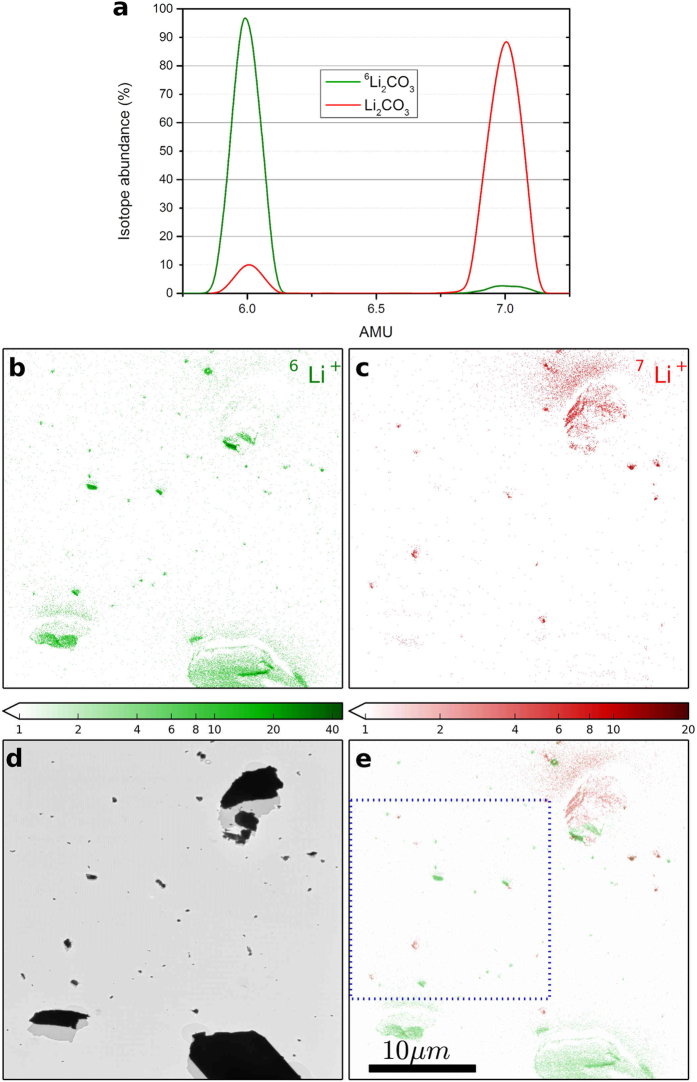
(**A**) Mass spectra obtained from samples containing only natural Li_2_CO_3_ (in red) and only isotopically enriched variant ^6^Li_2_CO_3_ (in green) (**B**) SIMS image of ^6^Li^+^ (**C**) SIMS image of ^7^Li^+^ (**D**) BF- TEM image of the same area and (**E**) Overlay of ^6^Li^+^ and ^7^Li^+^ secondary ion distributions. Note some smearing of SIMS intensity in front of large particles in (**B**,**C**) resulting from re-deposition of sputtered material (cf. Fig. 3A). The color scales indicate secondary ion counts in log scale. The area highlighted in blue is analyzed in more detail in Fig. 4.

The powder samples of natural and isotopically-enriched lithium carbonate were procured from Sigma Aldrich: (i) natural Li_2_CO_3_ (product code 13010, natural abundance of ^6^Li: 7.5 at. %) and (ii) ^6^Li_2_CO_3_ (product code 473111, specified isotopic enrichment of ^6^Li: 95 at. %). By integrating the area under the peaks in the mass spectra shown in Fig. 2, the local abundance of ^6^Li was experimentally found to be 8.9% and 96.8% for the samples (i) and (ii) respectively, which is in good agreement with the expected values globally. Sample (iii) described in the main text is a physical mixture of the samples (i) and (ii). The nanoparticles were suspended in ethanol and sonicated to obtain a good dispersion. A drop of the suspension was then deposited on conventional Cu-grids for TEM with amorphous carbon support membrane and the sample was then transferred to the PIES instrument.

## Additional Information

**How to cite this article**: Yedra, L. *et al*. In-situ Isotopic Analysis at Nanoscale using Parallel Ion Electron Spectrometry: A Powerful New Paradigm for Correlative Microscopy. *Sci. Rep*. **6**, 28705; doi: 10.1038/srep28705 (2016).

## Supplementary Material

Supplementary Information

Supplementary Video S1

## Figures and Tables

**Figure 1:**
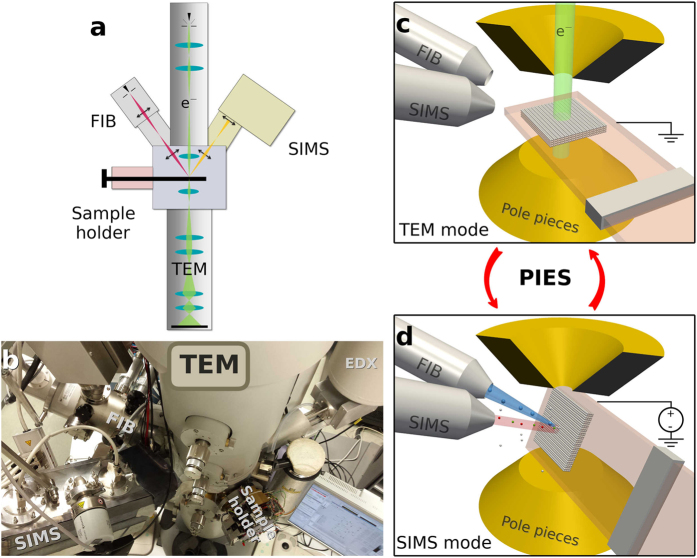
Schematics and a photo of the PIES instrument combining TEM, FIB and SIMS *in-situ*.A video illustration of the methodology is available in the Supplements.

**Figure 3:**
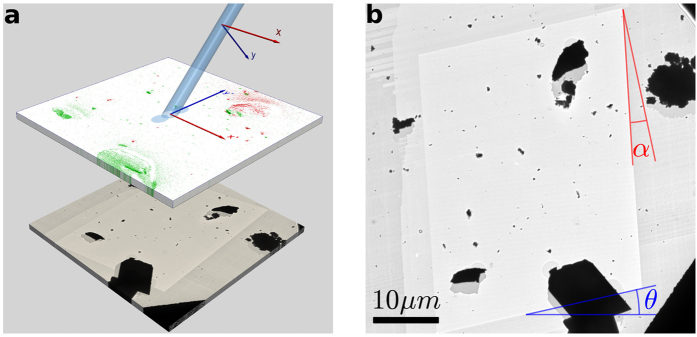
(**A**) Schematic of the configuration of the primary ion beam with respect to the sample surface. Note that a square raster in the primary ion beam coordinates will transform into a rectangle on the sample-coordinates due to geometric projection. Some re-deposited sputtered material in y′ direction can be seen in the SIMS overlay image. To illustrate the relative orientation of the TEM image with respect to the primary ion beam, the TEM image is shown below the SIMS image. (**B**) A lower magnification TEM image of the area investigated by SIMS directly reveals the size and shape of the area analyzed by SIMS. With such TEM images, the angular differences due to image-rotation (θ) and shear (α) can be directly and rapidly obtained.

**Figure 4:**
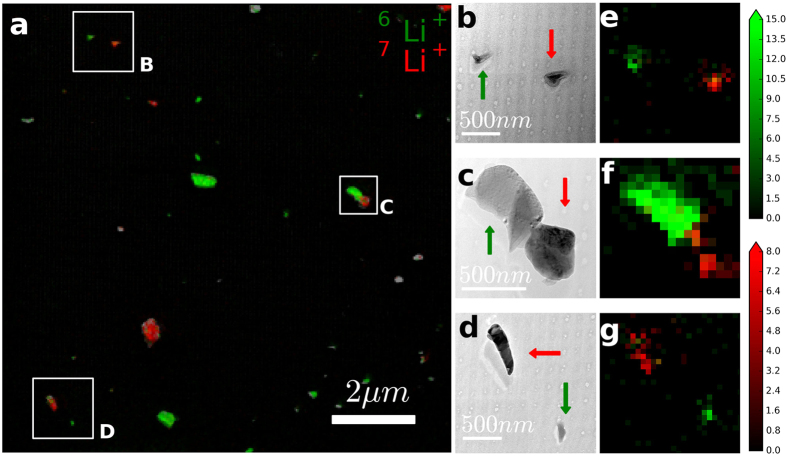
(**A**) PIES image: Overlay of BF-TEM image (contrast inverted) and SIMS images of ^6^Li^+^ and ^7^Li^+^, (**B–D**) high-magnification TEM images (contrast as acquired) corresponding to the boxed hotspots and (**E–G**) SIMS images of ^6^Li^+^ (green) overlaid on ^7^Li^+^(red) corresponding to images B–D respectively. The arrows in the TEM images indicate nanoparticles rich in ^6^Li^+^ (green) and ^7^Li^+^ (red). The color scales indicate secondary ion counts in linear scale.

## References

[b1] Nelson C. J. & Millar A. H. Protein turnover in plant biology. Nature Plants 1, 15017 (2015). 27246884 10.1038/nplants.2015.17

[b2] Angelo M. . Multiplexed ion beam imaging of human breast tumors. Nat Med 20, 436–442 (2014). 24584119 10.1038/nm.3488 PMC4110905

[b3] Steinhauser M. L. . Multi-isotope imaging mass spectrometry quantifies stem cell division and metabolism. Nature 481, 516–519 (2012). 22246326 10.1038/nature10734 PMC3267887

[b4] Lechene C. P., Luyten Y., McMahon G. & Distel D. L. Quantitative Imaging of Nitrogen Fixation by Individual Bacteria Within Animal Cells. Science 317, 1563–1566 (2007). 17872448 10.1126/science.1145557

[b5] Zhang D. S. . Multi-isotope imaging mass spectrometry reveals slow protein turnover in hair-cell stereocilia. Nature 481, 520–524 (2012). 22246323 10.1038/nature10745 PMC3267870

[b6] Sheik A. R. . *In situ* phenotypic heterogeneity among single cells of the filamentous bacterium Candidatus Microthrix parvicella. ISME J 10, 1274–1279 (2016). 26505828 10.1038/ismej.2015.181 PMC5029219

[b7] Cabin-Flaman A. . Combed Single DNA Molecules Imaged by Secondary Ion Mass Spectrometry. Anal. Chem. 83, 6940–6947 (2011). 21851091 10.1021/ac201685t

[b8] Wild E. M. . Direct dating of Early Upper Palaeolithic human remains from Mladec. Nature 435, 332–335 (2005). 15902255 10.1038/nature03585

[b9] Stolarski J., Meibom A., Przenioslo R. & Mazur M. A Cretaceous Scleractinian Coral with a Calcitic Skeleton. Science 318, 92–94 (2007). 17916731 10.1126/science.1149237

[b10] Shewmon P. G. Diffusion in solids (McGraw-Hill, New York, 1963).

[b11] De Souza R. A. & Martin M. Secondary ion mass spectrometry (SIMS) - a powerful tool for studying mass transport over various length scales. Phys. Status Solidi (c) 4, 1785–1801 (2007).

[b12] Landolt D. Corrosion and surface chemistry of metals (EPFL Press, 2007).

[b13] Debaille V., Brandon A. D., O’Neill C., Yin Q. Z. & Jacobsen B. Early martian mantle overturn inferred from isotopic composition of nakhlite meteorites. Nature Geosci 2, 548–552 (2009).

[b14] Floss C. . Carbon and Nitrogen Isotopic Anomalies in an Anhydrous Interplanetary Dust Particle. Science 303, 1355–1358 (2004). 14988560 10.1126/science.1093283

[b15] Wacey D., Kilburn M. R., Saunders M., Cliff J. & Brasier M. D. Microfossils of sulphur-metabolizing cells in 3.4-billion-year-old rocks of Western Australia. Nature Geosci 4, 698–702 (2011).

[b16] Misra S. & Froelich P. N. Lithium Isotope History of Cenozoic Seawater: Changes in Silicate Weathering and Reverse Weathering. Science 335, 818–823 (2012). 22282473 10.1126/science.1214697

[b17] Yeung L. Y., Ash J. L. & Young E. D. Biological signatures in clumped isotopes of O_2_. Science 348, 431–434 (2015). 25908819 10.1126/science.aaa6284

[b18] Lozada-Hidalgo M. . Sieving hydrogen isotopes through two-dimensional crystals. Science 351, 68–70 (2016). 26721995 10.1126/science.aac9726

[b19] Hüger E. . Lithium Transport through Nanosized Amorphous Silicon Layers. Nano Lett. 13, 1237–1244 (2013). 23360370 10.1021/nl304736t

[b20] Lucas M. S., Günthert M., Gasser P., Lucas F. & Wepf R. Chapter 17 - Bridging Microscopes: 3D Correlative Light and Scanning Electron Microscopy of Complex Biological Structures In Methods in Cell Biology. Correlative Light and Electron MIcroscopy (ed. Müller-Reichert Thomas & Verkade Paul) 325–356 (Academic Press, 2012). 10.1016/B978-0-12-416026-2.00017-0 22857936

[b21] Burnett T. L. . Correlative Tomography. Scientific Reports 4, 4711 (2014). 24736640 10.1038/srep04711 PMC3988479

[b22] Benninghoven A., Werner H. W. & Rüdenauer F. G. Secondary ion mass spectrometry: Basic concepts, instrumental aspects, applications and trends (Wiley-Interscience, New York, Chichester, Brisbane, Toronto, Singapore, 1987).

[b23] Wirtz T., Philipp P., Audinot J. N., Dowsett D. & Eswara S. High-resolution high-sensitivity elemental imaging by secondary ion mass spectrometry: from traditional 2D and 3D imaging to correlative microscopy. Nanotechnology 26, 434001 (2015). 26436905 10.1088/0957-4484/26/43/434001

[b24] Orloff J., Swanson L. W. & Utlaut M. Fundamental limits to imaging resolution for focused ion beams. J. Vac. Sci. Technol. B 14, 3759–3763 (1996).

[b25] Wirtz T. . Towards secondary ion mass spectrometry on the helium ion microscope: An experimental and simulation based feasibility study with He+ and Ne+ bombardment. Appl. Phys. Lett. 101, 041601–041605 (2012).

[b26] Egerton R. F., McLeod R., Wang F. & Malac M. Basic questions related to electron-induced sputtering in the TEM. Ultramicroscopy 110, 991–997 (2010).

[b27] Williams D. B. & Carter C. B. Transmission Electron Microscopy - A Textbook for Materials Science (Springer, US 2009).

[b28] Egerton R. Electron Energy-Loss Spectroscopy in the Electron Microscope (Springer, US 2011).

[b29] Hoppe P., Cohen S. & Meibom A. NanoSIMS: Technical Aspects and Applications in Cosmochemistry and Biological Geochemistry. Geostand Geoanal Res 37, 111–154 (2013).

[b30] Meijering E. H. W., Niessen W. J. & Viergever M. A. Quantitative evaluation of convolution-based methods for medical image interpolation. Medical Image Analysis 5, 111–126 (2001). 11516706 10.1016/s1361-8415(00)00040-2

[b31] Pavlic G., Singhroy V., Duk-Rodkin A. & Alasset P. J. Satellite Data Fusion Techniques for Terrain and Surficial Geological Mapping. Geoscience and Remote Sensing Symposium, IGARSS, IEEE International 3, 314–317 (2008).

[b32] Rangarajan S. & Tyler B. J. Topography in secondary ion mass spectroscopy images. J. Vac. Sci. Technol. A 24, 1730–1736 (2006).

[b33] Kraft M. L., Weber P. K., Longo M. L., Hutcheon I. D. & Boxer S. G. Phase Separation of Lipid Membranes Analyzed with High-Resolution Secondary Ion Mass Spectrometry. Science 313, 1948–1951 (2006). 17008528 10.1126/science.1130279

[b34] Migeon H. N., Saldi F., Gao Y. & Schuhmacher M. Ion microscope and ion microprobe analysis under oxygen, cesium and gallium bombardment. International Journal of Mass Spectrometry and Ion Processes 143, 51–63 (1995).

[b35] Philipp P., Wirtz T., Migeon H. N. & Scherrer H. SIMS analysis with neutral cesium deposition: Negative secondary ion sensitivity increase and quantification aspects. Int. J. Mass Spectrom. 253, 71–78 (2006).

[b36] Philipp P., Wirtz T., Migeon H. N. & Scherrer H. Important increase of negative secondary ion sensitivity during SIMS analysis by neutral cesium deposition. Appl. Surf. Sci. 252, 7205–7207 (2006).

[b37] Frache G., Adib B. E., Audinot J. N. & Migeon H. N. Evaluation of ionization yields under gallium bombardment. Surf. Interface Anal. 43, 639–642 (2011).

[b38] Pillatsch L. . Study and optimisation of SIMS performed with He+ and Ne+ bombardment. Appl. Surf. Sci. 282, 908–913 (2013).

