# Supplementary material for: In-situ Isotopic Analysis at Nanoscale using Parallel Ion Electron Spectrometry: A Powerful New Paradigm for Correlative Microscopy
Source: Sci Rep. 2016 Jun 28;6:28705. doi: 10.1038/srep28705 (PMC4923888; doi:10.1038/srep28705)
Supplement: Supplementary Information [file srep28705-s1.pdf]

Supplementary Materials for

In-situ Isotopic Analysis at Nanoscale using Parallel Ion Electron Spectrometry: A Powerful New Paradigm for Correlative Microscopy

Lluís Yedra, Santhana Eswara*, David Dowsett and Tom Wirtz

Corresponding author: santhana.eswara@list.lu

This PDF file includes:

Figure S1
Caption for Movie S1

Other Supplementary Materials for this manuscript includes the following:

Movie S1

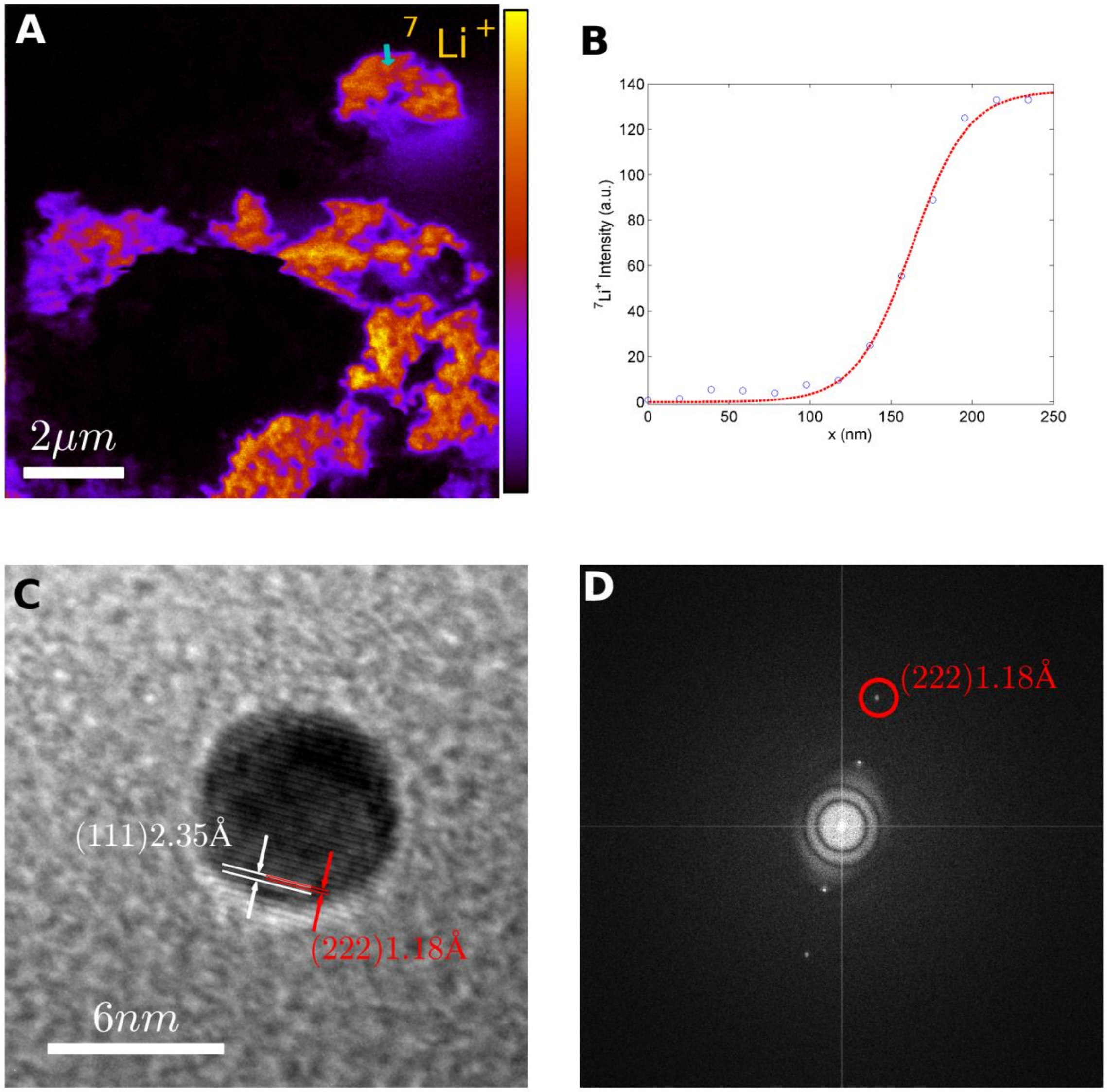

Fig. S1.

(A) SIMS image of $^7Li^+$ in the PIES instrument from lithium titanate sample acquired with a primary Ga^+ ion current of 100 pA, (B) line-profile corresponding to the small blue arrow in (A) with 16-84% intensity rise spanning over just below 60 nm, (C) High resolution TEM image of a gold nanoparticle showing (111) lattice planes and (D) FFT of image (C) showing spatial frequencies of up to (222) planes indicating that the lattice resolution of sub-1.5 Å can be obtained in the TEM mode (200 kV).

Movie S1

Video of PIES schematics.
